# Supplementary material for: Augmented tactile-perception and haptic-feedback rings as human-machine interfaces aiming for immersive interactions
Source: Nat Commun. 2022 Sep 5;13:5224. doi: 10.1038/s41467-022-32745-8 (PMC9445040; doi:10.1038/s41467-022-32745-8)
Supplement: Supplementary file 1 — Supplementary Information [file 41467_2022_32745_MOESM1_ESM.pdf]

Supplementary Information

Augmented Tactile-Perception and Haptic-Feedback Rings as Human-Machine Interfaces Aiming for Immersive Interactions

Zhongda Sun, [1,2,3] Minglu Zhu, [1,2,4] Xuechuan Shan, [3,5] and Chengkuo Lee [1,2,3,4,6,*]

[1] Department of Electrical & Computer Engineering, National University of Singapore, 4 Engineering Drive 3, 117583, Singapore

[2] Center for Intelligent Sensors and MEMS, National University of Singapore, 4 Engineering Drive 3, 117583, Singapore

[3] Singapore Institute of Manufacturing Technology and National University of Singapore (SIMTech-NUS) Joint Lab on Large-area Flexible Hybrid Electronics, National University of Singapore, 4 Engineering Drive 3, Singapore 117583, Singapore.

[4] National University of Singapore Suzhou Research Institute (NUSRI), Suzhou Industrial Park, Suzhou 215123, China

[5] Printed Intelligent Device Group, Singapore Institute of Manufacturing Technology, Agency for Science, Technology and Research (A*STAR), Singapore 637662, Singapore

[6] NUS Graduate School-Integrative Sciences and Engineering Program (ISEP), National University of Singapore, Singapore 119077, Singapore

[*] E-mail: (C. L.) elelc@nus.edu.sg

Supplementary Notes

Supplementary Note 1 | Working mechanism of the TENG tactile sensor.

The basic working mechanism of the TENG bending sensor is based on the coupling of contact electrification and electrostatic induction[1]. When two materials with different electron affinities contact and separate, surface charges will be generated on the contact surface due to triboelectrification and held on the surface, which will result in the varying electrical potential in the attached electrodes in the following contact-separation circles, generating the current flow in the external load and the triboelectric outputs.

Supplementary Note 2 | Working mechanism of the pyroelectric temperature sensor.

For the working mechanism of the pyroelectric temperature sensor, the output is generated based on the thermally induced random swing of the electric dipole around its balance axis in the pyroelectric material[2]. As shown in Supplementary Fig. 2, When the polarized pyroelectric film contact with the hot object, the spread of the electric dipoles on their respective alignment axes becomes greater, reducing the spontaneous polarization and resulting in the electron flow due to the electrostatic induction, thus generating the outputs.

Supplementary Note 3 | Structure optimization of the TENG tactile sensor.

As shown in Supplementary Fig. 4a, the influences of the pyramid dimensions on outputs are presented. It is found that silicone rubber films with the biggest pyramid dimensions (W1=3mm, W2=0.75mm) and lowest pyramid intensities can generate the largest outputs. The reason for the above phenomenon may be the difference in detecting the concentrated area of the finger muscle deformation. The lower the pyramid density, the larger area of the major muscle deformation can be covered by the more concentrated pyramid array. In addition, as depicted in Supplementary Fig. 4b and 4c, the size of the TPU ring and the

pyramid height are also factors that affect the outputs. In Supplementary Fig. 4b, the maximum output is achieved with a ring diameter of 20 mm and a pyramid height of 3 mm. While in Supplementary Fig. 4c, for the finger with the same size, the maximum output can be achieved when the TPU ring diameter becomes 22 mm and the pyramid height is 4 mm. The above results show that a suitable tightness can contribute to an optimal output voltage. Considering the differences in human finger sizes in one hand, differentiated ring sizes and pyramid heights may be needed for the optimal sensor output of each finger.

Supplementary Note 4 | Advantages of voltage integration based signal processing method for TENG tactile sensors.

To highlight the advantages of voltage integration based signal processing method, we draw a small table that compares the state-of-art TENG-based strain sensors for finger motion tracking in terms of sensing mechanism, quantization method, resolution, measurement tool/platform and gesture recognition application as shown in Supplementary Table 3. Based on the information from previous works, if we want to achieve continuous tracking based on TENG sensors, we either use a measuring instrument with extremely large internal resistance, i.e., electrometer, to obtain an approximate open-circuit measurement environment to detect the open-circuit voltage or transferred charge quantity, or use a grating-sliding structural sensor to measure the deformation/displacement based on the number of generated peaks. For methods based on the open-circuit measurement environment, the instruments are commonly bulky and expensive, which are not suitable for daily usage and wearable/portable application scenarios. While for the detection method based on the grating-sliding mode sensors, though the signal could be collected by the commercial or customized ADC on portable measurement platforms, the resolution is limited by the size and spacing of the grating electrodes, and is difficult to reach a very high level without advanced fabrication processes, e.g., MEMS process, screen printing, etc., which means high fabrication cost. Besides, even if the

size/spacing of the gating can be further reduced, the distinguishability of the signal will be a concern. Essentially, the measurement method based on the grating-sliding mode is not completely continuous because it requires electrodes to be arranged intermittently with certain gaps, where the size/spacing of the grading electrodes determines how much information is lost. As listed in Supplementary Table 3, the current best resolution could be achieved by grating-sliding mode TENG sensor for finger motion tracking is 3.8°. However, in this work, the continuous changes of the muscles during finger bending can be well reflected in the deformation of the pyramid structure to generate the corresponding continuous output as depicted in Fig. 2 and Supplementary Fig. 5, making the resolution can be as low as 1°.

In addition, the proposed voltage integration method can reflect this continuous signal change in a portable platform only by coding or algorithm, without the need for bulky and expensive open-circuit measuring instruments, providing the possibility to realize the real continuous measurement of TENG sensor signal on the mobile terminal, which have not be achieved by previous works (Supplementary Table 3).

Another advantage of using the voltage integration method is the interpretation capability of continuous gestures. Current TENG-based gesture recognition works all use the load voltage as the input signal for gesture recognition as listed in Supplementary Table 3, where the motion of making a specific gesture will be influenced by the gesture of the previous moment, resulting in the difference in gesture signals between the single gesture, and the same gesture in a continuous sentence. This phenomenon is explained clearly in the main text of the article and Fig. 3f. Though some works (ref 22, 26, 12 in Supplementary Table 3) have successfully realized continuous gesture recognition by analyzing the load voltage signal of a whole sentence, this method is not suitable for practical applications considering the various combinations of sentences in sign language and the time and labor costs required to build such a database. However, for our proposed voltage integration method, due to that there is nearly no difference in voltage integration signals between the single gesture and the corresponding

gesture in a sentence, we can just build a data set based on the single gesture, which can help us greatly save the cost of collecting gestures, and improve the universality of the data set for continuous sign language interpretation.

In summary, the signal processing approach based on the voltage integration signal for TENG-based sensory systems shows some advantages over previous methods based on the load voltage amplitude or grating-sliding peak numbers, for continuous stimuli/motion monitoring on portable platforms, thanks to the simple and lightweight measurement environment, i.e., customized ADC. Between, it also shows merits in the application scenarios of continuous sign language or gestures.

Supplementary Note 5 | Signal normalization process of the TENG tactile sensor to ensure generalization in practical applications**.**

For actual applications, it's impractical to provide a custom ring size for every finger of every user. A more practical approach is that we provide several sizes from small to large to meet the needs of different users as much as possible, in which case the signal differences are inevitable. The best solution to this issue is to use a software algorithm to calibrate the sensor signal based on each user's finger size on the first use. As shown in Supplementary Fig. 7, different finger sizes will result in the variation of sensor sensitivity with the same size of ring and pyramid structure. However, due to the approximately linear relationship between the bending angle and the output, if we regard the green line in the figure as the standard output of the sensor, then we can easily convert the output of different fingers by a factor to the standard output during calibration. This can be counted as a simple normalization process, which is also a common way to avoid individual errors when doing machine learning[3]. Similarly, pulse-like signals can also be easily normalized based on output amplitude. As long as all data, both collected in real-time and in the database, are normalized, the errors between individuals can be avoided and will not influence the accuracy of identification. This

calibration process only needs to be performed the first time the user uses it and does not affect the ease of use of the device. This method avoids the complexity of calibration at the hardware level and greatly reduces the cost of the targeted design.

Supplementary Note 6 | Power consumption and efficiency of the overall system.

Power consumption efficiency is a quite important parameter for smart devices, especially wearable/portable devices, considering the limited lifespan of batteries and the annoying replacement and recharging process. Because our system is mainly composed of sensing and feedback units, so this problem is mainly discussed from three aspects: sensor, vibro-haptic feedback unit and thermo-haptic feedback unit: 1) In our system, the TENG tactile sensor and PVDF temperature sensor are all based on the nanogenerator that can convert the energy from the human body or in the ambient into the signal, and do not need the power supply. Compared to current commercial wearable sensors whose power consumption is in the range of 3-300 μW[4], the self-powered sensors in our system are undoubtedly more energy efficient. 2) For the vibro-haptic feedback unit, based on the comparison of different vibrators in the recent related work[5], the ERM vibrator can induce larger vibration intensity under the same operation power compared to other types of vibrators, i.e., linear resonance actuator (LRA), piezoelectric actuator and voice coil actuator[6]. This can help to create a larger and more robust feedback sensation more efficiently. Between, compared to other vibrators that need to vibrate at a fixed resonant frequency, the ERM vibrator changes both the vibration frequency and amplitude under different supply voltages, which introduces a stronger change of feeling for users[7]. 3) For thermo-haptic feedback devices, we have drawn a table to compare the state-of-art works for wearable scenarios (Supplementary Table 5), including the heating technologies based on the Joule heating and thermoelectric. As listed in Supplementary Table 5, our solution based on the NiCr wire can provide a somatosensory temperature close to

55 °C under the power supply voltage of 1.8 V, which shows good energy efficiency compared to other related works.

Between, we have also tested the power consumption of each unit in our system, including the vibrator, heater, signal processing unit and data communication module (Supplementary Table 6). Because our sensors are self-powered, so they can be counted as zero-power components and the power consumption of the integrated wearable system mainly comes from the signal processing unit, wireless data communication module and haptic feedback units (vibrators and heaters). As for machine learning, optimization systems and VR systems, these are all carried out through IoT on local terminals or cloud with supercomputing power and external power supply. In this case, the power consumption generated by these functions is less important to our wearable system. The power consumption and the number of each unit have been listed in Supplementary Table 6, where the power consumption of the haptic units accounts for the main part. Considering the maximum power consumption of the actuators in the actual application scenario, the peak overall power consumption can be calculated as 1.95 W, where all vibrators are at maximum vibration intensity and the heater is heated to around 55 °C when attached to the skin. As for the application scenarios that do not need haptic feedback functions, the overall power consumption is around 0.25 W, which is quite low for real-time HMIs with high sampling speed thanks to the self-powered sensors utilized in the system.

When compared with other similar HMIs with multifunctional sensing and haptic-feedback capabilities, because the overall functions of the integrated system are not completely consistent, we cannot use a unified standard to measure and compare the overall energy efficiency. However, as mentioned above, the sensing and feedback units in our system have good energy efficiency when taken out individually and compared with other works, so the overall energy efficiency of the system should also be at a good level.

Supplementary Note 7 | Mutual interference test between the haptic feedback units and the TENG tactile sensor.

In Supplementary Fig. 15, we compare the outputs of the TENG tactile sensor collected by our customized IoT module when the vibrator was not actuated (Supplementary Fig. 15a) and at maximum vibration intensity (Supplementary Fig. 15b). It is clear that though the noise density increases a little bit under the maximum vibration intensity, the maximum amplitude of the noise doesn't change. So the effect of noise can be removed with a threshold of the same size without affecting the output of the sensor considering the huge difference in amplitude between the noise (maximum amplitude: 0.005 V) and the valid signal. The interference from the vibration could be ignored in our system mainly because we have added an RC filter circuit (low pass filter: < 10 Hz) in the signal processing circuit (see Supplementary Fig. 3), where the hundred-hertz noise from vibrations can be easily filtered out.

Supplementary Fig. 16 shows the influence of the temperature change on the TENG tactile sensor output. In our design, although the tactile sensor and heater are integrated into one unit, the two parts are separate and spaced apart internally as illustrated in Supplementary Fig. 16a. When the heater is heated to more than 70 °C, though the sensing area will be affected by the temperature due to heat diffusion, the maximum temperature of the sensing part is about 40 °C as shown in Supplementary Fig. 16b. In Supplementary Fig. 16c, we test the TENG tactile sensor outputs when the temperature of the working environment increases from 25 °C to 45 °C, and the result shows that the TENG output will not experience significant fluctuation under such working conditions. In our daily use, the temperature of the required thermal feedback will not be too high. In this case, the temperature change generated by the heater will not have too much influence on the output of the sensor.

Supplementary Note 8 | Working mechanism of the HTV “Lighthouse” tracking system.

The trackers (Supplementary Fig. 19a) and headset use a positional tracking system known as "Lighthouse", where multiple external base station units (Supplementary Fig. 19b) are installed in the play area, which each contain an array of LED lights, and two infrared lasers. The lasers are attached to rotating spinners which sweep the play area vertically and horizontally with timed pulses. The headset and trackers contain photosensors that detect the LED lights from the base stations, and then compare them with the timing of the laser sweeps in order to determine their relative position in 3D space.

Supplementary Note 9 | The response time of the multimodal sensing and feedback platform for metaverse**.**

In our metaverse-based interactive system depicted in Fig. 5a, the response process can be divided into two parts. One is the object recognition part, where the object in the real space of a user could be projected into the virtual space on the cloud through the collected sensor signals and machine learning. Another is the real-time control and haptic feedback part, where the user can control the motion of the virtual hand in the metaverse space and feel the virtual object via the vibro- and thermo-haptic feedback functions. The process of the object recognition part is shown in detail in Supplementary Fig. 20a, mainly including the acquisition of tactile and temperature signals when grasping objects, the wireless transmission in the local area network, the real-time object recognition based on machine learning, and the communication with the server twice. Based on the response time of the TENG sensor, PVDF sensor, actuators, wireless transmission, ML recognition and cloud server shown in Supplementary Fig. 20c-d and Supplementary Table 7, we know that the response time of the signal acquisition process is determined by the PVDF sensor due to its longer response time compared to that of the TENG tactile sensor. The total response time of the object recognition part can be calculated as ~ 176 ms. While for the real-time control and haptic feedback part shown in Supplementary Fig. 20b, the whole process contains the acquisition of tactile signals,

the wireless transmissions in the local area network twice, and the actuation of vibro- and thermo-haptic feedback units, where the response time of the feedback actuation process is mainly determined by the heater. Because the response time of the heater is determined by the driving power and the maximum temperature to be reached. Therefore, in the case of the highest driving power of the platform, and considering the temperature (< 60 °C) to be reached in practical applications, the ideal response time of the real-time control and haptic feedback part could be calculated as ~ 9.13 s. If we combine these two parts into an interactive system, the time for a complete interaction is ~ 9.3 s, which is acceptable considering the generally long response time of the thermal feedback function.

Supplementary Figures

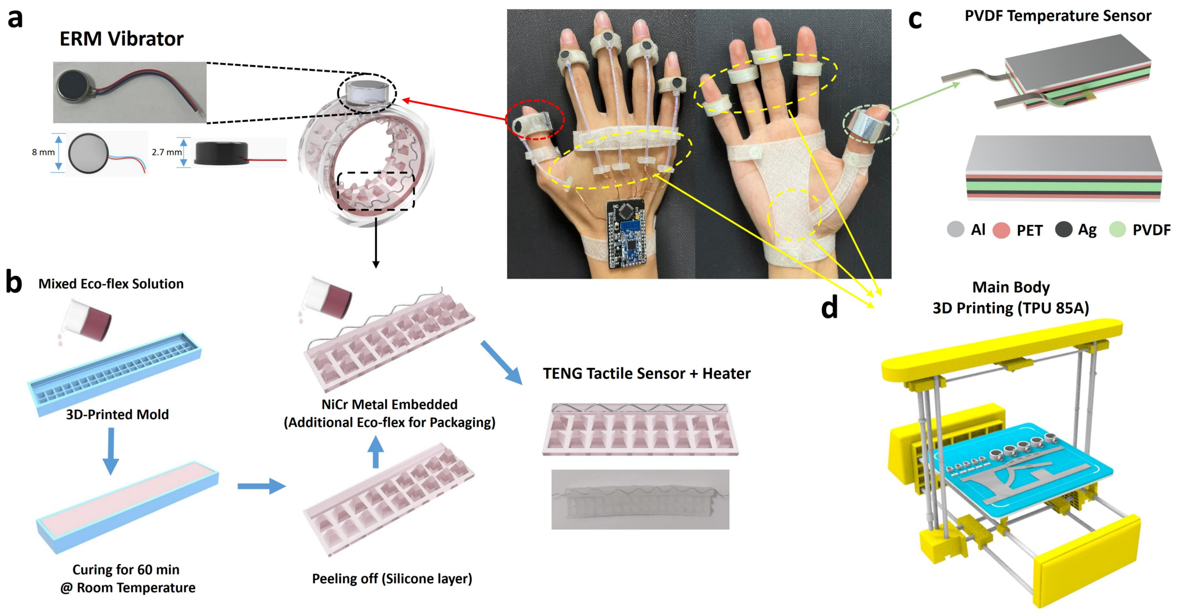

Supplementary Fig. 1 | The illustration of the overall system and the structure/fabrication processes of different components. **a,** Photo of the ERM vibrator with labeled dimension. **b,** The detailed fabrication process of the TENG tactile sensor with the NiCr metal wire heater. **c,** The structure of the PVDF temperature sensor. **d,** Illustration of the 3D printing process of soft components for connection and assembly.

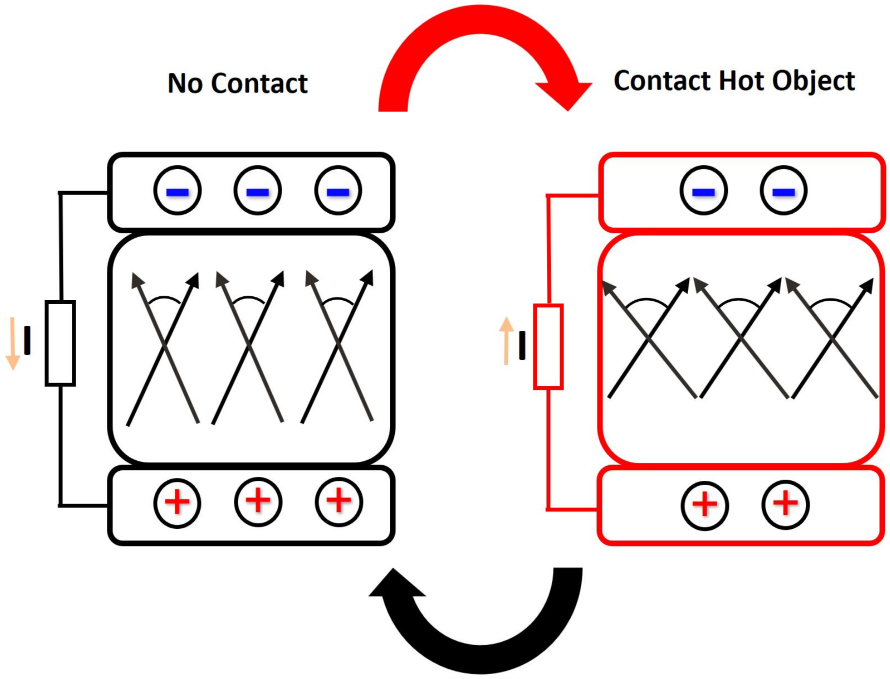

Supplementary Fig. 2 | Working mechanism of the pyroelectric sensor for temperature sensing.

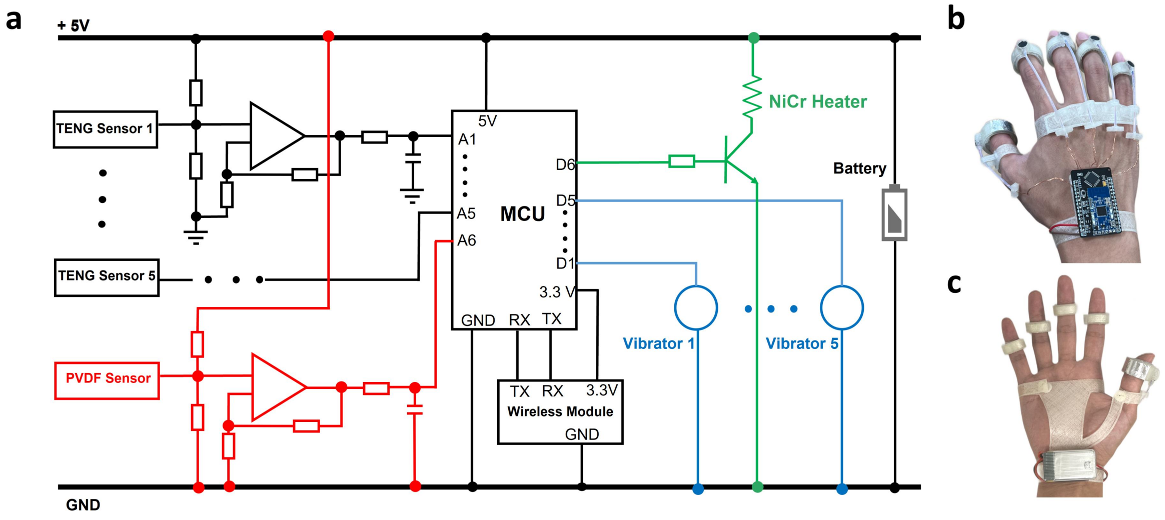

Supplementary Fig. 3 | The Architecture of the entire system. **a**, The detailed circuit schematic shows all connections between the sensing/feedback units and the IoT module. The illustration of the integrated system with the **b**, IoT module (front side) and **c**, power supply battery (back side).

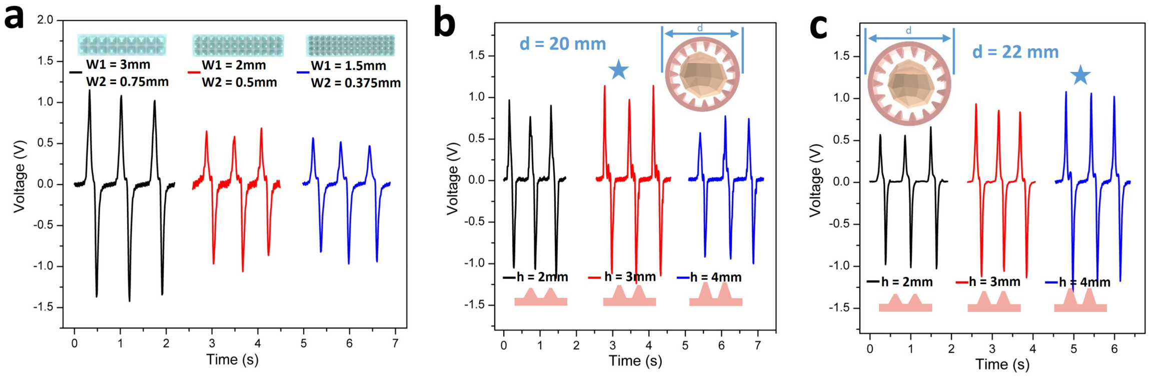

Supplementary Fig. 4 | The output optimization of the TENG tactile sensor in terms of different **a,** pyramid sizes and **b-c,** ring diameters and pyramid heights.

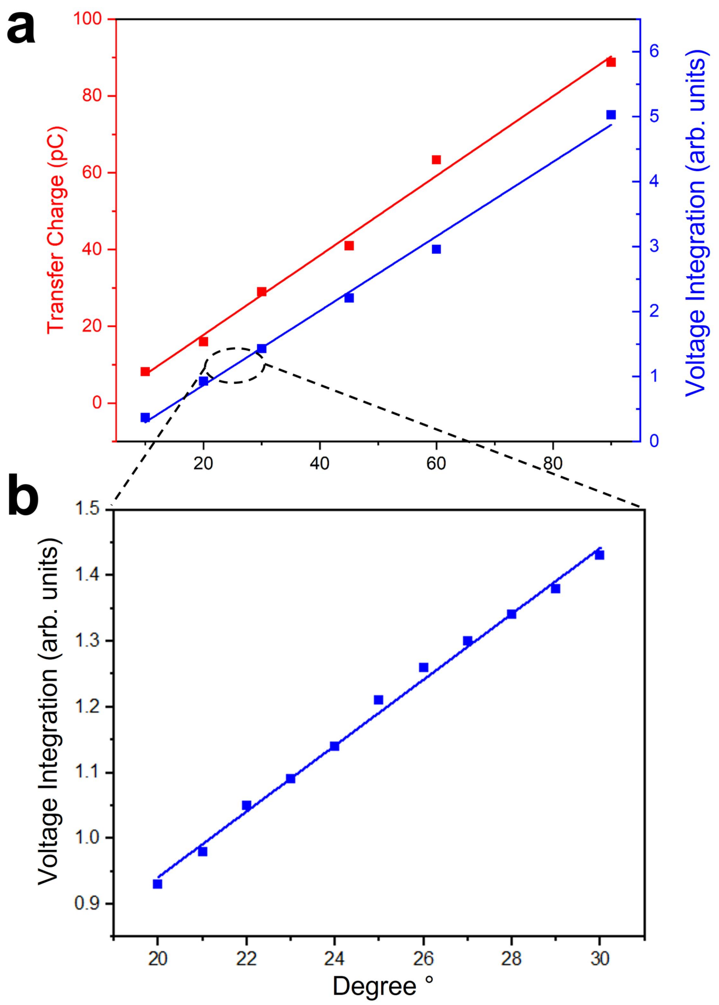

Supplementary Fig. 5 | a, The transfer charge and the voltage integration output of the TENG tactile sensor under different finger bending angles. **b,** The voltage integration output of the TENG tactile sensor when the bending angle is increased from 20° to 30° in 1° interval.

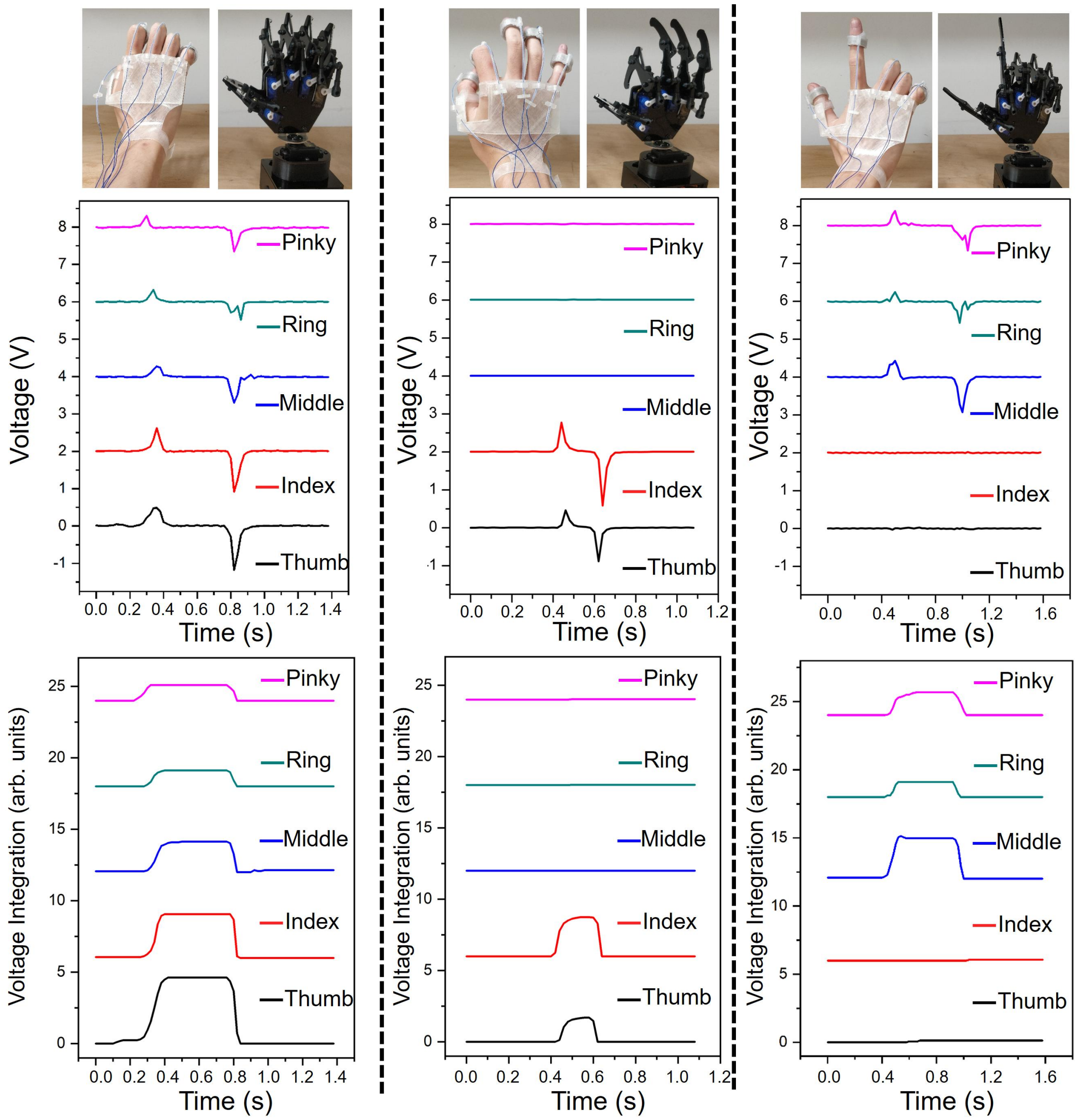

Supplementary Fig. 6 | The TENG tactile sensor outputs and the corresponding motion illustrations for multi-finger gesture control.

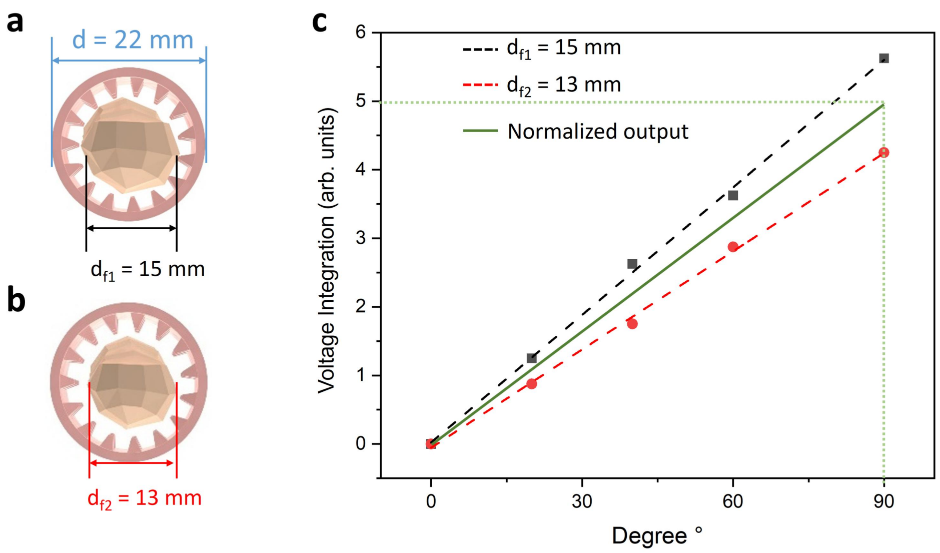

Supplementary Fig. 7 | Signal normalization process of the TENG tactile sensor. Illustrations of the TENG tactile sensor when the diameters of the finger are **a,** 15 mm and **b,** 13 mm, respectively, with fixed ring size (22 mm) and pyramid height (3 mm). **c,** The corresponding voltage integration outputs in terms of different bending angles and the normalized signal output to avoid individual differences.

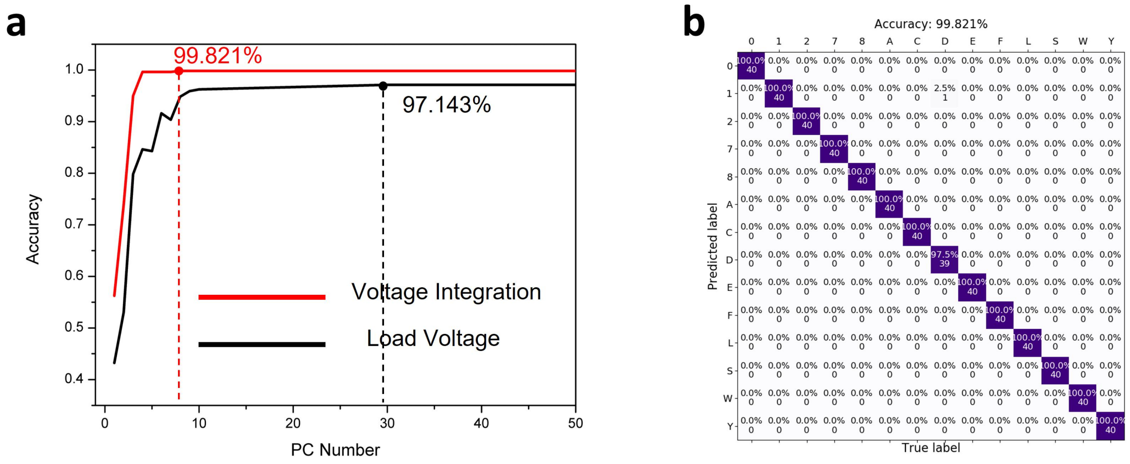

Supplementary Fig. 8 | Merits of voltage integration method for gesture/sign language recognition. **a,** The relationship between the accuracy and the number of principal components used for two data formats: voltage integration and load voltage. **b,** The confusion map for the data set only containing the final stable values of the integral signals for the 14 sign language gestures.

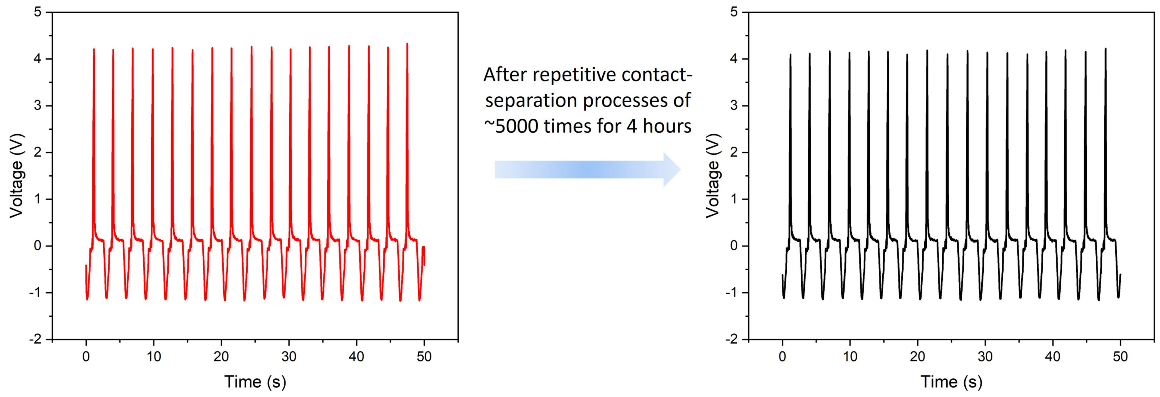

Supplementary Fig. 9 | Durability test of the TENG tactile sensor after 4 hours of repetitive contact-separation process at 40 N.

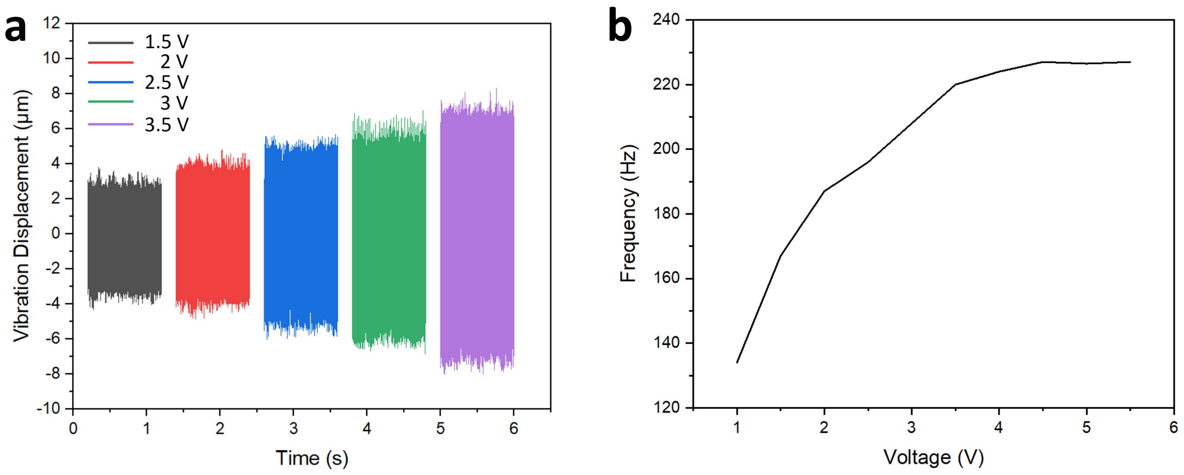

Supplementary Fig. 10 | The actual **a,** vibration amplitudes and **b,** frequencies of the ERM vibrator under different supply voltages.

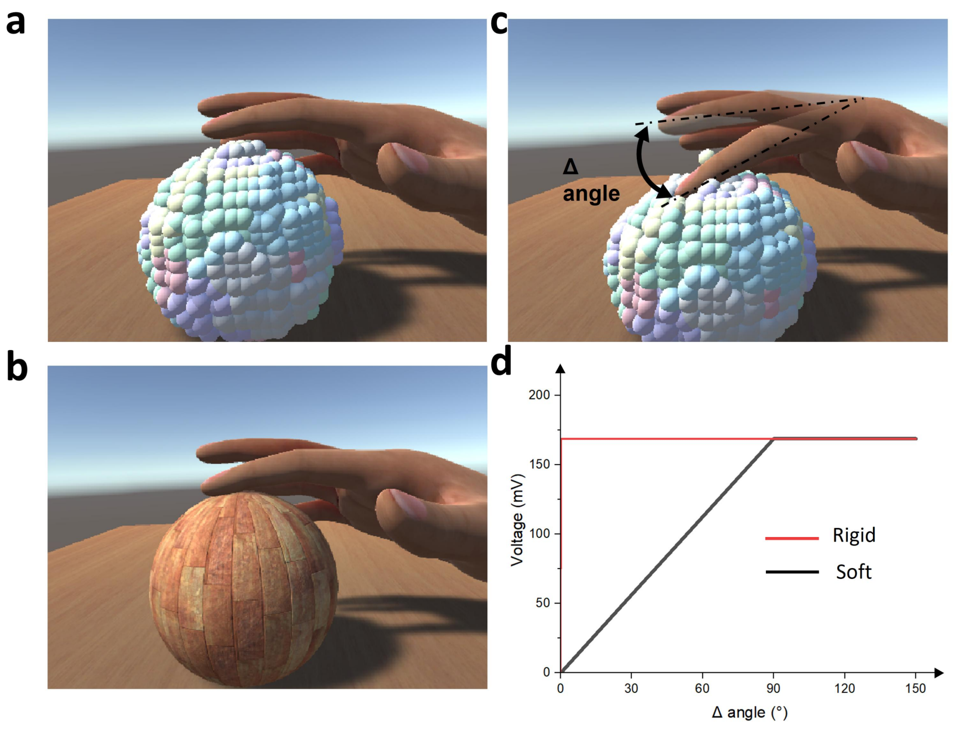

Supplementary Fig. 11 | Control logic of vibration intensity for touching rigid and soft virtual objects. The illustrations of pressing **a,** soft and **b,** rigid virtual objects. **c-d,** The relationship between squeezing/pressing angle and vibration intensity for virtual objects with different stiffness.

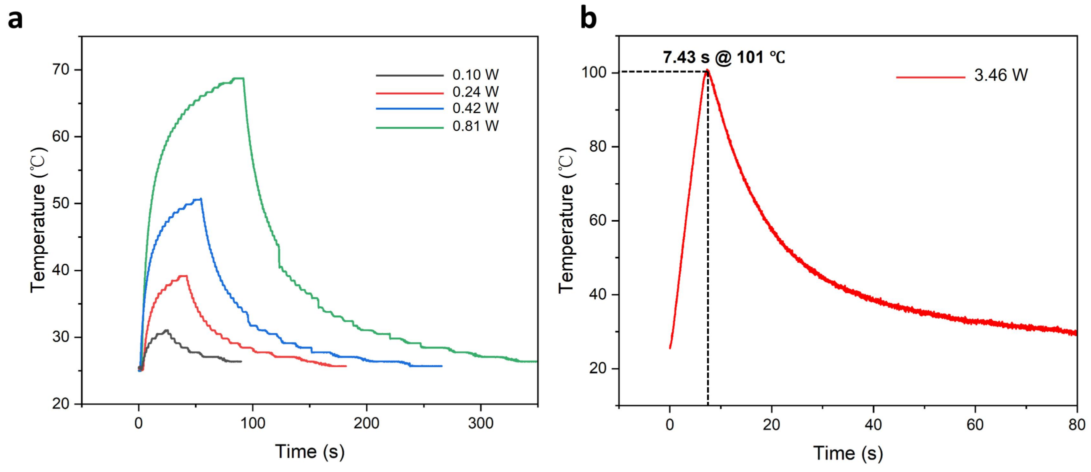

Supplementary Fig. 12 | The response time of the thermo-haptic feedback unit when placed on a TPU substrate, with another side exposed to the air. **a,** The response time of the heater corresponding to different driven power. **b,** The rapid heating curve of the heater for fast response scenario, 101 °C within 7.43 s with driven power of ~3.5 W.

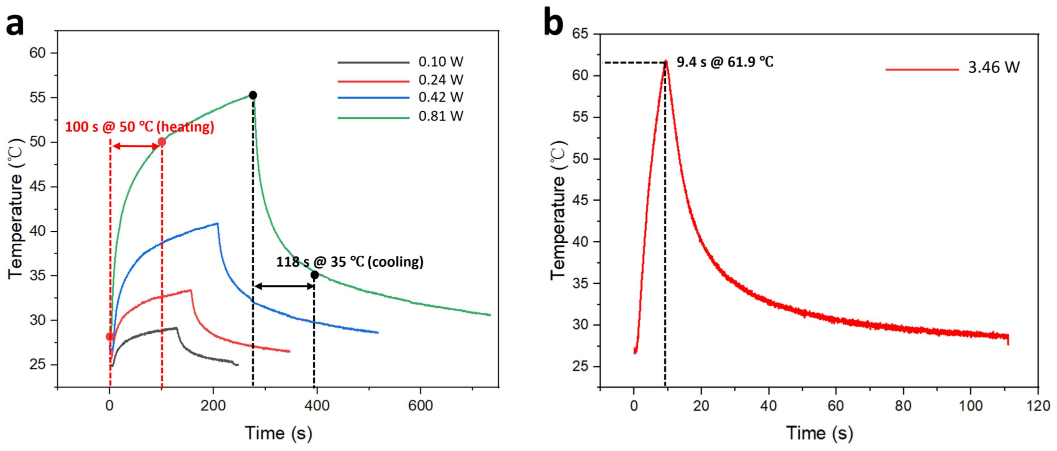

Supplementary Fig. 13 | The response time of the thermo-haptic feedback unit when placed on a TPU substrate, with another side in contact with skin. a, The temperature response of the contacting interface corresponding to different driven power. b, The rapid heating curve of the contacting interface for fast response scenario, 61.9 °C within 9.4 s with driven power of ~3.5 W.

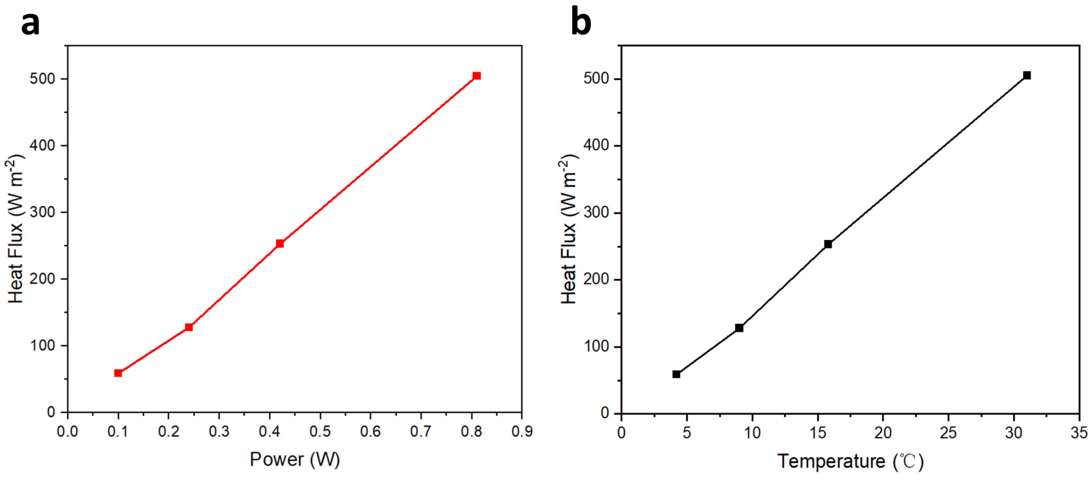

Supplementary Fig. 14 | The heat flux of the thermo-haptic feedback unit when placed on a TPU substrate, with another side in contact with skin. a, The heat flux of the heating interface under different driven power. b, The relationship between the temperature and heat flux of the heating interface.

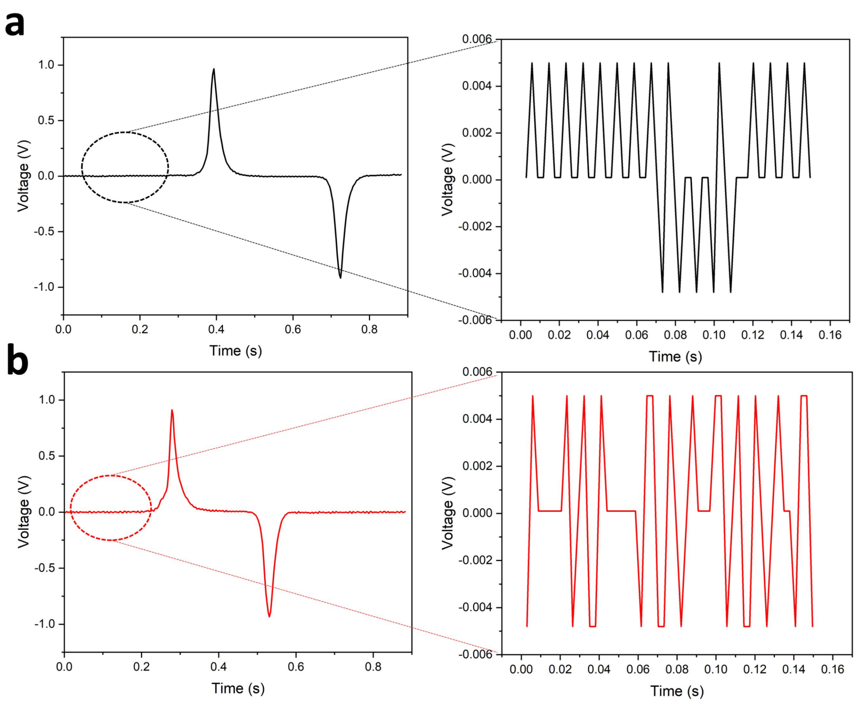

Supplementary Fig. 15 | The outputs of the TENG tactile sensor collected by our customized IoT module when the vibrator **a,** is not actuated and **b,** at maximum vibration intensity.

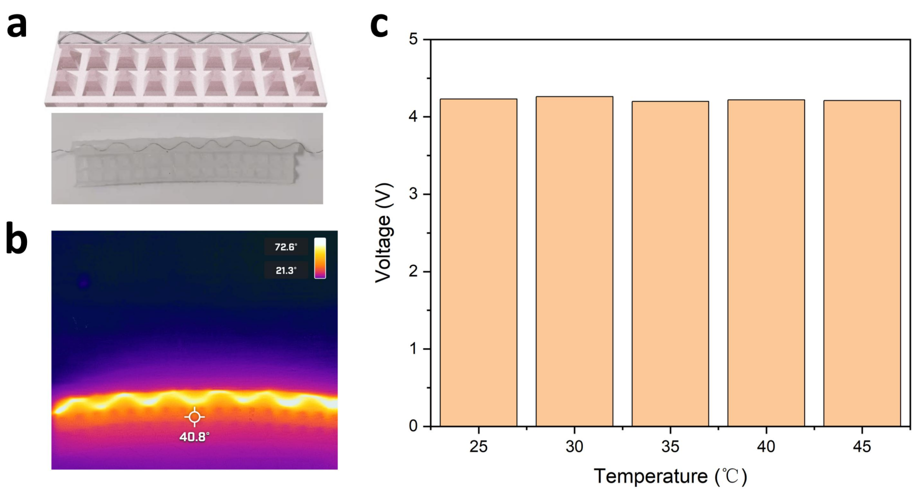

Supplementary Fig. 16 | The influence of the temperature change on the TENG tactile sensor output. **a,** Illustration of the TENG tactile sensor with the NiCr metal wire heater. **b,** The maximum temperature of the tactile sensing area when the heater is heated to more than 70 °C. **c,** The outputs of the TENG tactile sensor under the consistent pressure of 40 N when the temperature of the sensor increases from 25 °C to 45 °C.

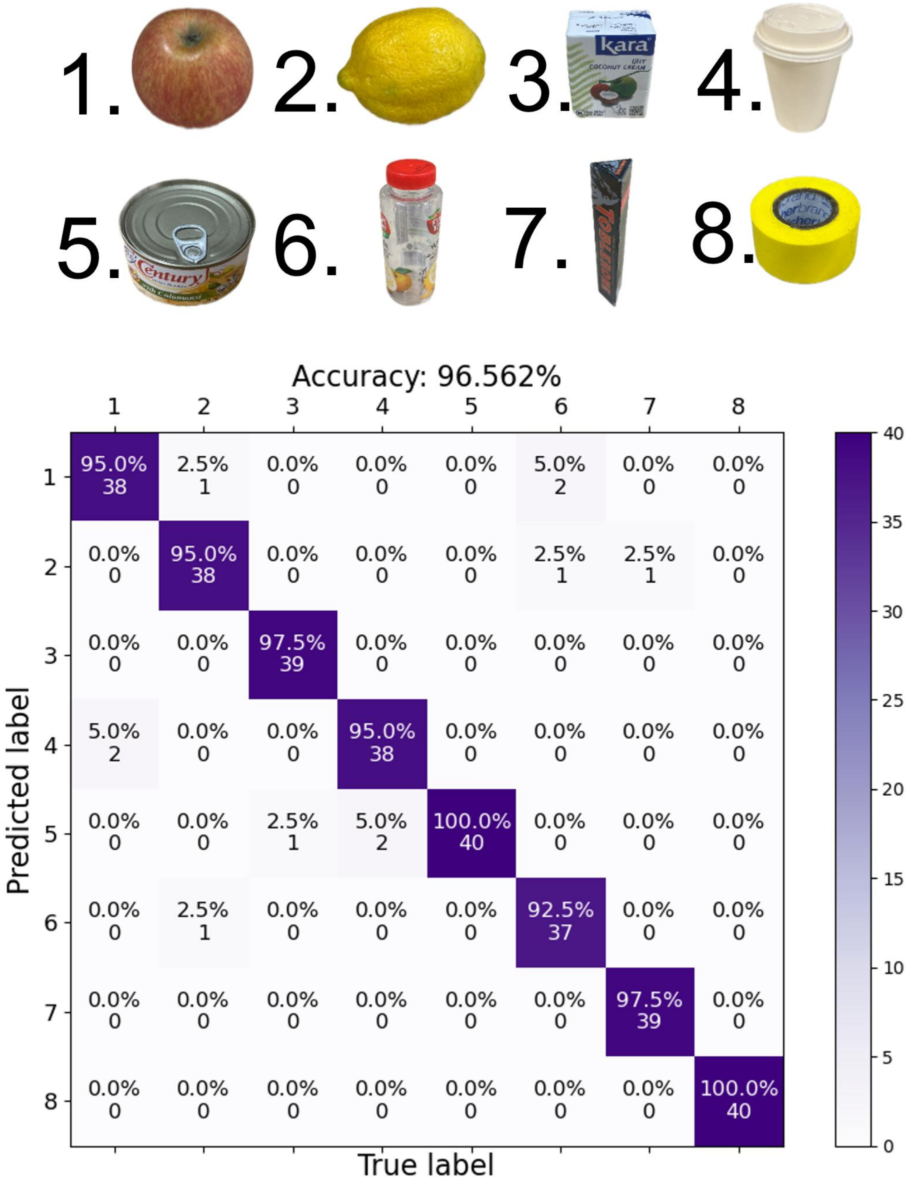

Supplementary Fig. 17 | Illustration and the confusion map for 8 common daily items used for grasped object recognition.

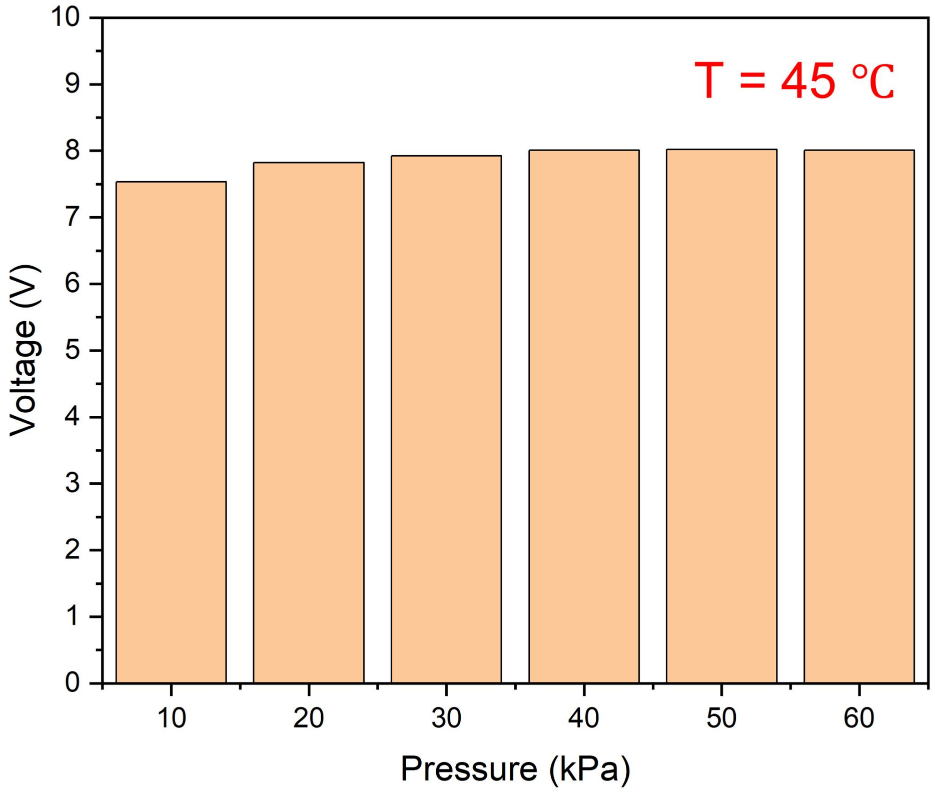

Supplementary Fig. 18 | The influence of pressure on temperature sensor output by fixing the temperature of the touched object at 45 ℃.

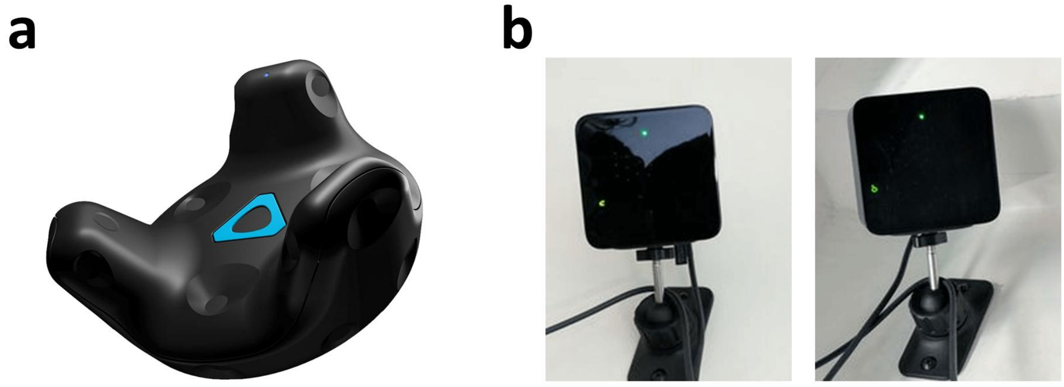

Supplementary Fig. 19 | Photos of the components for HTC tracking system, including the **a,** trackers and **b,** base station units.

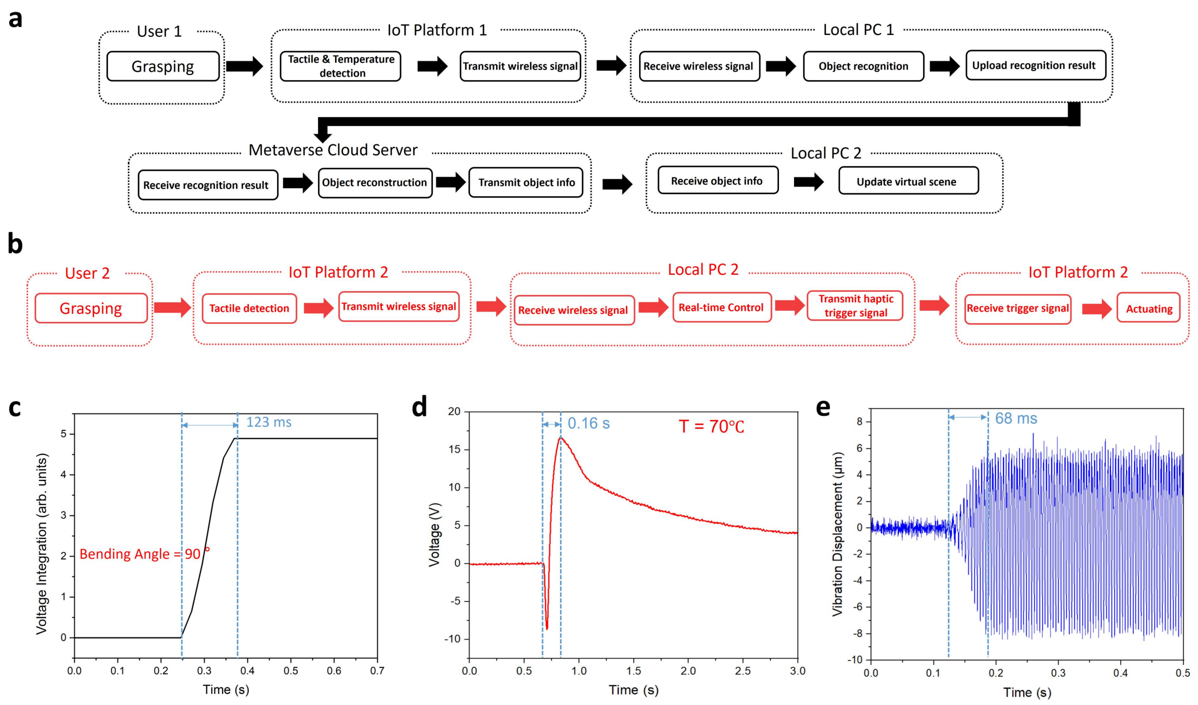

Supplementary Fig. 20 | The response time of the multimodal sensing and feedback platform for metaverse. The response process of the **a,** object recognition part and **b,** real-time control and haptic feedback part. The response time of the **c,** TENG tactile sensor, **d,** PVDF temperature sensor and **e,** ERM vibrator respectively.

Supplementary Tables

Supplementary Table 1 | Comparison of state-of-art glove-based HMIs.

Ref	Type	Sensing			Feedback		Fully Portable System
		Tactile	Temperature	Self-powered	Tactile	Temperature	
8	Glove	Carbon-nanotube-based resistive strain sensor	NA	No	NA	NA	NA
9	Glove	Liquid-metal-based resistive pressure sensor	NA	No	NA	NA	NA
3	Glove	Conductive-copolymer-based resistive pressure sensor	NA	No	NA	NA	NA
10	Glove	Yarn-based triboelectric strain sensor	NA	Yes	NA	NA	Yes
11	Glove	Fiber-optic-based strain/pressure sensor	NA	No	NA	NA	NA
12	Glove	Triboelectric textile strain sensor	NA	Yes	NA	NA	NA
13	Glove	Flexible piezoelectric strain sensor	NA	Yes	Pneumatic actuator actuated by electrostatic attraction	NA	No (Input voltage > 1kV for actuator)
14	Glove	NA	NA	NA	Tendon-Driven actuation system	NA	No (Big Actuator)
15	Glove	NA	NA	NA	Electrostatic clutches	NA	No (Input voltage ≈300V for actuator)
16	Glove	Flexible piezoelectric strain sensor	NA	Yes	NA	Thermoelectric device	Yes
17	Glove	Silicone-based triboelectric pressure sensor	NA	Yes	Piezoelectric-based vibrator	NA	NA
18	Glove	Liquid-metal-based resistive strain sensor	NA	No	Vibrator	Electroresistive heater	NA

[19]	Ring	NA	NA	NA	Dielectric elastomer actuators	NA	Yes
**This Work**	**Ring**	**Silicone-based triboelectric strain sensor**	**PVDF temperature sensor**	**Yes**	**ERM Vibrator**	**NiCr metal resistive heater**	**Yes**

Supplementary Table 2 | The technical parameters of 3D printing.

Quality	
Layer height (mm)	0.1
Shell thickness (mm)	1.6
Enable retraction	No

Fill	
Bottom/Top thickness (mm)	2
Fill Density (%)	0

Speed and Temperature	
Print speed (mm/s)	20
Printing temperature (℃)	240
Bed temperature (℃)	25

Filament	
Diameter (mm)	1.2
Flow (%)	100

Machine	
Nozzle size (mm)	0.4

Supplementary Table 3 | Comparison of TENG-based strain sensors for finger motion tracking.

Ref	Sensing Mechanism	Materials	Continuous Tracking	Resolution	Measurement tool/platform	Gesture Recognition
20	Grating-Sliding Mode (Peak Number)	PDMS/Water	Yes	45°	Oscilloscope	NA
21	Contact-Separation Mode (Charge Quantity)	FEP/Al	Yes	NA	Electrometer	NA
22	Contact-Separation Mode with Interlocked Structure (Voltage Amplitude)	P(VDF-TrFE)/PDMS	No	10°	Oscilloscope	Limited Continuous Gesture Recognition
23	Contact-Separation Mode with Yarn-based Structure (Voltage Amplitude)	Conductive yarn/ Silicone rubber	No	NA	Electrometer	Single Gesture
24	Contact-Separation Mode with Ladder-shaped Structure (Voltage Amplitude)	PTFE/Al	No	20°	Electrometer	Single Gesture
25	Grating-Sliding Mode (Peak Number)	FEP/Copper	Yes	3.8°	Commercial ADC	NA
26	Contact-Separation Mode with E-textile Based Sensor (Voltage Amplitude)	Nylon/Polyeste r	No	20°	Customized ADC	Limited Continuous Gesture Recognition
27	Contact-Separation Mode with Arch-shaped Structure (Voltage Amplitude)	PEDOT:PSS coated textile/Silicone rubber	No	30°	Customized ADC	NA
28	Contact-Separation Mode with Arch-shaped Structure (Voltage Amplitude)	CNT-TPE coated textile/Silicone rubber	No	30°	Customized ADC	Single Gesture
29	Contact-Separation Mode with Nestable Arch-shaped Structure (Open-circuit Voltage)	Silicone rubber/Al	Yes	NA	Electrometer	NA
30	Contact-Separation Mode with Microstructured Layers (Voltage Amplitude)	Nylon/PTFE	No	15°	Customized ADC	Single Gesture
10	Contact-Separation Mode with Yarn-based Structure (Voltage Amplitude)	PDMS/Polyest er	No	NA	Customized ADC	Single Gesture
17	Contact-Separation Mode with Dome-shaped Sensor (Voltage Amplitude)	Silicone rubber	No	30°	Customized ADC	Single Gesture
31	Contact-Separation Mode (Voltage Amplitude)	Silicone rubber/PDMS	No	1°	Customized ADC	Single Gesture
32	Grating-Sliding Mode with Magnetic Array (Peak Number)	FEP/Copper	Yes	18°	Commercial ADC	NA
12	Contact-Separation Mode with Textile-based Sensor	Silicone rubber/Nitrile	No	NA	Customized ADC	Limited Continuous Gesture

	(Voltage Amplitude)					Recognition
**This work**	**Contact-Separation Mode with Pyramid-structural Layer (Voltage Integration)**	**Silicone rubber**	**Yes**	**1°**	**Customized ADC**	**Enhanced Continuous Gesture Recognition**

Supplementary Table 4 | Comparison of wearable sensory systems for gesture/object recognition.

Ref	Type	Sensing Type		ML-enabled Function		Based on Self-powered Sensing Mechanism
		**Tactile**	**Strain**	**Object Recognition**	**Gesture Recognition**	
3	Glove	Piezoresistive sensor array (548 sensors)	NA	≈84% (26 objects)	89.4% (8 gestures)	No
28	Glove	NA	Triboelectric textile sensor (10 sensors)	NA	95.23% (11 gestures)	Yes
17	Glove	Triboelectric dome-shaped tactile sensor (16 sensors)	NA	96.88% (6 objects)	NA	Yes
33	Glove	Fluidic pressure sensor (6 sensors)	Resistive knit sensor (16 sensors)	99.7% (30 objects)	NA	No
10	Glove	NA	Triboelectric yarn-shaped sensor array (5 sensors)	NA	98.63% (11 gestures)	Yes
34	Sleeve	NA	Resistive sensor array (4 sensors)	NA	88.29% (3 gestures)	No
35	E-skin	NA	Resistive skin-like sensor (1 sensor)	NA	96.2% (8 finger motions)	No
36	E-skin	NA	sEMG electrode arrays on flexible substrates (16 x 4 array)	NA	97.12% (13 gestures) 92.87% (21 gestures)	No
12	Glove	Triboelectric textile sensor (3 sensors)	Triboelectric textile sensor (10 sensors)	NA	91.3% (50 sign language words)	Yes

**This work**	**Ring**	**NA**	**Triboelectric ring-shaped sensor (5 sensors)**	**96.56% (8 daily objects)**	**99.82% (14 gestures)**	**Yes**

Supplementary Table 5 | Comparison of state-of-art thermo-haptic feedback technologies for wearable scenarios.

Ref	Type	Mechanism	Material	Power Supply	Wearability	Heating/Cooling
37	E-skin	Joule Heating	Ag nanowire	2.8 - 6.2 V @ 50 ℃	Stretchable & Flexible	Heating
38	Wrist	Joule Heating	Ag nanowire	3.7 V @ 37 ℃ (Skin)	Stretchable & Flexible	Heating
39	Glove	Joule Heating	Cu nanowire	4 V @ 60 ℃	Stretchable & Flexible	Heating
40	Sleeve	Joule Heating	Carbon nanotube fiber	7 V @ 48 ℃ (Skin)	Flexible	Heating
41	Sleeve	Joule Heating	Carbon nanotube fiber	9 V @ 42 ℃ (Skin)	Flexible	Heating
42	Glove	Joule Heating	PEDOT/PSS fiber	9 V @ 36 ℃ (Skin)	Flexible	Heating
43	Electronic tattoos	Joule Heating	Semi-liquid-metal Ni-EGaIn	0.4 A @ 45.7 ℃ (Skin)	Stretchable & Flexible	Heating
44	Kneepad	Joule Heating	Liquid-metal galinstan	2 V @ 50 ℃	Stretchable & Flexible	Heating
18	Glove	Joule Heating	Liquid-metal eGaIn	1 W @ 80 ℃	Stretchable & Flexible	Heating
45	Vest	Thermoelectric	Bi_2Te_3	180 mW @ △ 6 ℃ (Skin)	Flexible (Thickness ≈ 6 mm)	Cooling
46	Patch	Thermoelectric	Bi_2Te_3	5 V 1.33 A @ △ 5 ℃ (Skin)	Flexible (Thickness ≈ 10 mm)	Cooling

47	Glove	Thermoelectric	Bi_2Te_3	0.6 A @ ~ 35℃<br>1.5 A @ ~ 12 ℃	Stretchable (Thickness ≈ 2 mm)	Heating & Cooling
**This work**	**Ring**	**Joule Heating**	**NiCr wire**	**0.45 A 1.8 V @ 55 ℃ (Skin)**	**Flexible**	**Heating**

Supplementary Table 6 | Power consumption of each unit in the system.

	Vibrator	Heater	Signal processing unit	Data communication module	Overall
Unit Number	5	1	1	1	
Power consumption (each unit)	0.18 W @ Maximum vibration intensity	0.81 W @ 55 ℃ (Skin)	0.09 W	0.15 W	1.95 W (Peak)

Supplementary Table 7 | Response time of different components in the system.

	TENG Sensor	PVDF Sensor	Vibrator	Heater	Wireless Transmission	ML Recognition	Cloud Server
Response Time	123 ms @ Fast bending 90°	160 ms @ T = 70℃	68 ms	9 s @ 60 ℃ (Skin)	4.8 ms	~ 1 ms	5 ms

Supplementary References

1. Fan, F.-R., Tian, Z.-Q. & Lin Wang, Z. Flexible triboelectric generator. *Nano Energy* **1**, 328–334 (2012).
2. Zi, Y. *et al.* Triboelectric-Pyroelectric-Piezoelectric Hybrid Cell for High-Efficiency Energy-Harvesting and Self-Powered Sensing. *Adv. Mater.* **27**, 2340–2347 (2015).
3. Sundaram, S. *et al.* Learning the signatures of the human grasp using a scalable tactile glove. *Nature* **569**, 698–702 (2019).
4. Gunawardhana, K. R. S. D., Wanasekara, N. D. & Dharmasena, R. D. I. G. Towards Truly Wearable Systems: Optimizing and Scaling Up Wearable Triboelectric Nanogenerators. *iScience* **23**, 101360 (2020).
5. Jung, Y. H. *et al.* A wireless haptic interface for programmable patterns of touch across large areas of the skin. *Nat. Electron.* (2022) doi:10.1038/s41928-022-00765-3.
6. Yu, X. *et al.* Skin-integrated wireless haptic interfaces for virtual and augmented reality. *Nature* **575**, 473–479 (2019).
7. Huang, H. *et al.* Experiment and investigation of two types of vibrotactile devices. in *2016 6th IEEE International Conference on Biomedical Robotics and Biomechatronics (BioRob)* vols 2016-July 1266–1271 (IEEE, 2016).
8. Suzuki, K. *et al.* Rapid-Response, Widely Stretchable Sensor of Aligned MWCNT/Elastomer Composites for Human Motion Detection. *ACS Sensors* **1**, 817–825 (2016).
9. Gao, Y. *et al.* Wearable Microfluidic Diaphragm Pressure Sensor for Health and Tactile Touch Monitoring. *Adv. Mater.* **29**, 1–8 (2017).
10. Zhou, Z. *et al.* Sign-to-speech translation using machine-learning-assisted stretchable sensor arrays. *Nat. Electron.* **3**, 571–578 (2020).
11. Bai, H. *et al.* Stretchable distributed fiber-optic sensors. *Science (80-.).* **370**, 848–852

(2020).

12. Wen, F., Zhang, Z., He, T. & Lee, C. AI enabled sign language recognition and VR space bidirectional communication using triboelectric smart glove. *Nat. Commun.* **12**, 5378 (2021).

13. Song, K. *et al.* Pneumatic actuator and flexible piezoelectric sensor for soft virtual reality glove system. *Sci. Rep.* **9**, 8988 (2019).

14. Kang, B. B., Choi, H., Lee, H. & Cho, K.-J. J. Exo-Glove Poly II: A Polymer-Based Soft Wearable Robot for the Hand with a Tendon-Driven Actuation System. *Soft Robot.* **6**, 214–227 (2019).

15. Hinchet, R. & Shea, H. High Force Density Textile Electrostatic Clutch. *Adv. Mater. Technol.* **5**, 1900895 (2020).

16. Kim, S.-W. *et al.* Thermal display glove for interacting with virtual reality. *Sci. Rep.* **10**, 11403 (2020).

17. Zhu, M. *et al.* Haptic-feedback smart glove as a creative human-machine interface (HMI) for virtual/augmented reality applications. *Sci. Adv.* **6**, eaaz8693 (2020).

18. Oh, J. *et al.* A Liquid Metal Based Multimodal Sensor and Haptic Feedback Device for Thermal and Tactile Sensation Generation in Virtual Reality. *Adv. Funct. Mater.* **31**, 2007772 (2021).

19. Ji, X. *et al.* Untethered Feel-Through Haptics Using 18-μm Thick Dielectric Elastomer Actuators. *Adv. Funct. Mater.* **31**, 1–10 (2021).

20. Shi, Q., Wang, H., Wang, T. & Lee, C. Self-powered liquid triboelectric microfluidic sensor for pressure sensing and finger motion monitoring applications. *Nano Energy* **30**, 450–459 (2016).

21. Jin, L., Tao, J., Bao, R., Sun, L. & Pan, C. Self-powered Real-time Movement Monitoring Sensor Using Triboelectric Nanogenerator Technology. *Sci. Rep.* **7**, 10521 (2017).

22. Ha, M. *et al.* Skin-Inspired Hierarchical Polymer Architectures with Gradient Stiffness for Spacer-Free, Ultrathin, and Highly Sensitive Triboelectric Sensors. *ACS Nano* **12**, 3964–3974 (2018).

23. Dong, K. *et al.* Versatile Core–Sheath Yarn for Sustainable Biomechanical Energy Harvesting and Real-Time Human-Interactive Sensing. *Adv. Energy Mater.* **8**, 1–12 (2018).

24. Lu, C. *et al.* A Stretchable, Flexible Triboelectric Nanogenerator for Self-Powered Real-Time Motion Monitoring. *Adv. Mater. Technol.* **3**, 1–8 (2018).

25. Pu, X. *et al.* Rotation sensing and gesture control of a robot joint via triboelectric quantization sensor. *Nano Energy* **54**, 453–460 (2018).

26. He, Q. *et al.* An all-textile triboelectric sensor for wearable teleoperated human-machine interaction. *J. Mater. Chem. A* **7**, 26804–26811 (2019).

27. He, T. *et al.* Self-powered glove-based intuitive interface for diversified control applications in real/cyber space. *Nano Energy* **58**, 641–651 (2019).

28. Wen, F. *et al.* Machine Learning Glove Using Self-Powered Conductive Superhydrophobic Triboelectric Textile for Gesture Recognition in VR/AR Applications. *Adv. Sci.* **7**, 2000261 (2020).

29. Liao, J. *et al.* Nestable arched triboelectric nanogenerator for large deflection biomechanical sensing and energy harvesting. *Nano Energy* **69**, 104417 (2020).

30. Maharjan, P. *et al.* A human skin-inspired self-powered flex sensor with thermally embossed microstructured triboelectric layers for sign language interpretation. *Nano Energy* **76**, 105071 (2020).

31. Luo, Y. *et al.* Triboelectric bending sensor based smart glove towards intuitive multi-dimensional human-machine interfaces. *Nano Energy* **89**, 106330 (2021).

32. Qin, K. *et al.* Magnetic Array Assisted Triboelectric Nanogenerator Sensor for Real-Time Gesture Interaction. *Nano-Micro Lett.* **13**, (2021).

33. Hughes, J. *et al.* A Simple, Inexpensive, Wearable Glove with Hybrid Resistive-Pressure Sensors for Computational Sensing, Proprioception, and Task Identification. *Adv. Intell. Syst.* **2**, 2000002 (2020).

34. Araromi, O. A. *et al.* Ultra-sensitive and resilient compliant strain gauges for soft machines. *Nature* **587**, 219–224 (2020).

35. Kim, K. K. *et al.* A deep-learned skin sensor decoding the epicentral human motions. *Nat. Commun.* **11**, 2149 (2020).

36. Moin, A. *et al.* A wearable biosensing system with in-sensor adaptive machine learning for hand gesture recognition. *Nat. Electron.* **4**, 54–63 (2021).

37. Hong, S. *et al.* Highly Stretchable and Transparent Metal Nanowire Heater for Wearable Electronics Applications. *Adv. Mater.* **27**, 4744–4751 (2015).

38. Choi, S. *et al.* Stretchable Heater Using Ligand-Exchanged Silver Nanowire Nanocomposite for Wearable Articular Thermotherapy. *ACS Nano* **9**, 6626–6633 (2015).

39. Kim, D. *et al.* Highly stretchable and oxidation-resistive Cu nanowire heater for replication of the feeling of heat in a virtual world. *J. Mater. Chem. A* **8**, 8281–8291 (2020).

40. Liu, P. *et al.* Stretchable and Energy-Efficient Heating Carbon Nanotube Fiber by Designing a Hierarchically Helical Structure. *Small* **14**, 1–6 (2018).

41. Luo, X. *et al.* Multifunctional fabrics of carbon nanotube fibers. *J. Mater. Chem. A* **7**, 8790–8797 (2019).

42. Zhou, J. *et al.* High-ampacity conductive polymer microfibers as fast response wearable heaters and electromechanical actuators. *J. Mater. Chem. C* **4**, 1238–1249 (2016).

43. Guo, R. *et al.* Semi-Liquid-Metal-(Ni-EGaIn)-Based Ultraconformable Electronic Tattoo. *Adv. Mater. Technol.* **4**, 1–11 (2019).

44. Wang, Y. *et al.* Printable Liquid-Metal@PDMS Stretchable Heater with High Stretchability and Dynamic Stability for Wearable Thermotherapy. *Adv. Mater. Technol.* **4**, 1–9 (2019).

45. Hong, S. *et al.* Wearable thermoelectrics for personalized thermoregulation. *Sci. Adv.* **5**, (2019).

46. Lee, D. *et al.* Liquid-metal-electrode-based compact, flexible, and high-power thermoelectric device. *Energy* **188**, 116019 (2019).

47. Lee, J. *et al.* Stretchable Skin-Like Cooling/Heating Device for Reconstruction of Artificial Thermal Sensation in Virtual Reality. *Adv. Funct. Mater.* **30**, 1–11 (2020).
