# Supplementary material for: Augmented tactile-perception and haptic-feedback rings as human-machine interfaces aiming for immersive interactions
Source: Nat Commun. 2022 Sep 5;13:5224. doi: 10.1038/s41467-022-32745-8 (PMC9445040; doi:10.1038/s41467-022-32745-8)
Supplement: Supplementary file 3 — Description of Additional Supplementary Files [file 41467_2022_32745_MOESM3_ESM.docx]

**Description of Additional Supplementary Files**

**File Name: Supplementary Movie 1
Description:** TENG tactile sensor performance.

**File Name: Supplementary Movie 2
Description:** Continuous robotic finger control.

**File Name: Supplementary Movie 3**

**Description:** Vibro-haptic feedback for VR application.

**File Name: Supplementary Movie 4**

**Description:** Virtual piano training application.

**File Name: Supplementary Movie 5**

**Description:** Thermohaptic feedback for VR application.

**File Name: Supplementary Movie 6**

**Description:** Interactive VR chat application.
